# The Birth Plan Experience—A Pilot Qualitative Study in Southern Spain

**DOI:** 10.3390/healthcare10010095

**Published:** 2022-01-04

**Authors:** Raquel Alba-Rodríguez, María Pilar Coronado-Carvajal, Pedro Hidalgo-Lopezosa

**Affiliations:** 1 Departamento de Enfermería, Farmacología y Fisioterapia, Universidad de Córdoba, 14004 Cordoba, Spain; raquelalba@outlook.com (R.A.-R.); en1cocam@uco.es (M.P.C.-C.); 2 Instituto Maimónides de Investigación Biomédica de Córdoba (IMIBIC), 14004 Cordoba, Spain

**Keywords:** birth plan, satisfaction, childbirth experience, prenatal care

## Abstract

Background: Healthcare systems advocate for quality care and humanized relations in routine birth care, and have therefore created the Birth Plan, a document available to pregnant women to state their preferences in relation to the birth process. Methods: This qualitative research with a phenomenological design was carried out to record the experiences of women who presented a Birth Plan. Sample selection was carried out using non-probabilistic, intentional and convenience sampling, selecting seven participants who were willing to participate and share their experiences. Results: After analyzing the content of the interviews, four categories emerged: “respecting the woman’s wishes: humanizing the birth process”, “information and primary Care”, “expectations regarding the care received” and “results of using the birth plan”, with their corresponding subcategories. Conclusion: Women consider it beneficial to present a Birth Plan, because it informs them about the process and gives them the opportunity to have a better experience, which takes into account their preferences for making the delivery less instrumental. In addition, they state the importance of having trained professionals involved, and call for more attention to be paid to the birth process in general.

## 1. Introduction

Labor is a female physiological process, and a major event and experience in the lives of women. Childbirth care has undergone significant changes over the years. Initially attended by traditional midwives at home, it moved to hospitals after the second half of the 20th century, leading to the medicalization of childbirth [1]. With this change to a hospital process, the mother began to be treated as a patient in need of medical attention, which has led to increased intervention during labor, resulting in the development of routine practices such as shaving, enemas, routine episiotomy, and others, often lacking in scientific evidence [2].

The Birth Plan (BP) is a document in which women can express their preferences, needs, desires and expectations regarding the birth process. However, this document in no way replaces the information provided by the medical staff, nor does it convert the mother into the main organizer of the labor process, but rather enables her to influence the aspects that are negotiable. These issues must be respected, provided the health of the mother or baby is not jeopardized [3].

Birth plans appeared for the first time in the United States at the end of the 1970s as a reaction to increasing medicalization, and as a consequence of women’s efforts to assert their preferences [4]. BPs can improve women’s satisfaction, promote their participation in the birth process, improve the communication between pregnant women and midwives, and help all parties to make informed decisions [2]. Some authors have found that using BPs may influence the mode of delivery, as it can improve obstetric results such as a lower cesarean rate [3,4,5,6]. However, BPs are not used by many women [7].

The World Health Organization includes the importance of effective communication in the recommendations published in 2018 for a positive birth experience. It focuses on experience of care as a critical aspect for ensuring high-quality labor, and improved woman-centered outcomes [8].

In Spain, the Ministry of Health and Social Policy published the ‘Estrategia de Atención al Parto Normal’ in 2009, which considered the BP as a useful document for pregnant women [9]. The preferences expressed in the BP involve the entire labor process, from the first stage of labor to the attention given to the newborn [10]; the most frequently-expressed preferences in the BP are to limit routine procedures such as oxytocin use or episiotomy, avoid cesarean section, permit freedom of movement, allow immediate contact with the newborn and early breastfeeding, among others [11]. However, the use of the BP may lead to certain conflicts between the women and the medical staff [2], generally caused by dissatisfaction due to their expectations not being fulfilled [9]. The fulfillment of and adherence to BPs is essential to improve women’s satisfaction [12,13]. Adherence to BPs is still quite low, and strategies of their implementation are therefore needed to increase the adoption of BPs as an important tool in every delivery [14]. Studies have shown an improvement in women’s perception of BPs and their adherence and compliance with them through the use of tools such as apps [15]. In general, BPs increase women’s level of satisfaction with the birth process and improve their experience of the delivery [11].

The aim of this study was to determine the impact of presenting a BP on the women’s personal experience, focusing on their expectations and the level of satisfaction reached.

## 2. Materials and Methods

Qualitative methodology and a phenomenological study design were used to obtain individual results through interviews with the participants [16]. The study was inductive and aimed to discover and interpret the experiences of women who presented a BP in a tertiary hospital in the Public Health System in Andalusia (Spain). Their experiences were described in detail, and a thorough analysis of the interviews was then made to understand these women’s experiences and feelings.

Sample selection was carried out by non-probabilistic, intentional and convenience sampling, with only those mothers who wished to collaborate and were willing to share their experiences and feelings being selected.

The size of the sample was determined by theoretical saturation. Throughout the process, we used the technique of snowball or chain sampling. Through contacts given by the first participants, we found other women who were willing to participate. Ultimately, 7 participants took part in the study. This sample, although small, was sufficient to address the thematic categories in the study. It should be taken into account that adherence to the birth plan was very low, both in the country and in the study setting itself, and that only about 5% of women were finally presented with a birth plan at the study hospital. The study also included women who had given birth in the three months prior to data collection and who had presented a BP. All the participants specifically expressed their interest in participating. The data was gathered during the months of February and March 2018.

To gather the data, semi-structured interviews were carried out with a pre-established script containing 11 questions, which were used flexibly depending on how the dialogue developed. Prior to this, the participants were given an informed consent sheet. The interviews lasted between 15 and 20 min and took place in a nurse’s consultation room in a health center, attended by all participants when taking part in a workshop on breastfeeding. All the sessions were audio recorded and, once transcribed, the recordings were deleted.

The data was processed by means of an analysis of the content involving four phases: transcription, reduction, codification and categorization, and all four categories contained closely-related subcategories (Table 1).

There is an established protocol on the use of BP. First, the primary care midwife provides information about the BP. Once the woman decides to present a BP to the referral hospital, the official model or a free handwritten one, a midwife from the hospital will arrange a meeting with the woman and her partner to inform them about the possibilities and limitations of the hospital’s services, as well as to show them the delivery room.

This study was approved by the provincial Ethics Committee (code TFGUCORAR). The participants were fully informed and all signed an informed consent sheet.

## 3. Results

### 3.1. Category 1: Respecting Wishes: Humanizing the Birth Process

The women filled in their BP to make the medical staff aware of what they considered important during their labor, and to state how they wanted to experience the birth, if circumstances allowed. Their aim was to humanize the process and experience it in the most natural way possible. This is how it was explained by participant number 7 (P7).

P7: “So that they would respect as much as possible how I wanted things to be done as regards my labor and the birth of my daughter; […] to make it more humane”.

#### Subcategory 1.1: A Means of Encouraging Better Communication

Regardless of the extent to which the BP was adhered to, the opinion of the participants was unanimous in that the BP was a tool that encouraged better communication between the midwife and user, and improved the birth experience. Without communication, their wishes could not be fulfilled and, in this, the midwife’s attitude was considered fundamental. Participant 2 expressed this as follows:

P2: “In my case, luckily for me, the midwife had read through everything point by point and we went over the BP together”. “If the staff who attend you are empathetic and receptive and have read the BP, then of course the plan facilitates the birth process”.

### 3.2. Category 2: Information and Primary Care

Most of the women valued the prenatal classes highly; however, they expressed the need for the staff to be up-to-date with the latest developments and for the classes to be coordinated well. They also expressed the importance of receiving information from specialized professionals, such as midwives. Although they were informed of the existence of the BP document, they stated that information about it was difficult to obtain, and they affirmed that they had used other sources to guide and inform them (internet, books, associations, family, and friends). Some participants responded as follows:

P3: “The information they give you is more or less okay […] but professional staff like midwives should be involved, as this is their field of work and they have more information”.

P6: “I think it was in the health center. Then I researched a lot into it myself”. “I asked in the health center if it was possible to visit the hospital facilities, but I got the impression that they didn’t have much up-to-date information and they didn’t know how to respond to many questions”.

### 3.3. Category 3: Expectations Regarding the Care Received

In general, the women expressed their satisfaction with the experience and with having presented a BP, and valued positively the fact that the medical staff were interested in their preferences. All the participants, apart from one, expressed a high degree of satisfaction.

P6: “For me, honestly, it was very positive […] everything was done in the way I had requested […] I was given a fair amount of freedom. The experience I had was very good”.

#### Subcategory 3.1. Demanding for Improvements Immediately after Birth

Some mothers requested a greater quality of care after birth and, in this regard, some participants expressed that they would value positively more guidance regarding breastfeeding or skin-to-skin contact.

P5: “Things as simple as skin-to-skin or some quick guidance on breastfeeding would have been appreciated... instead, everyone disappeared from the room. They didn’t help me with the issue of breastfeeding and then I had lots of problems”.

### 3.4. Category 4: Results of Using the Birth Plan

For most participants, the BP was useful beforehand as a guide and to give them an initial introduction to the experience of labor. However, two of the participants stated that it was of no use at all, as their expectations were not met.

P4: “For me yes. It helped me to know all of the options I had when giving birth, as I didn’t know of all of them […]”.

#### Subcategory 4.1: A Tool for Providing Legal Advice and Peace of Mind

The BP provided these mothers with greater peace of mind. The women were fully aware of their rights, and knew that the BP acted as an authentic ‘informed consent’ form, and that the medical staff were required to respect it as much as possible.

P1: “At that moment it gave me peace of mind […]; peace of mind, in the sense that if anything happens, the birth plan is there”.

## 4. Discussion

### 4.1. Category 1: Respecting the Women’s Wishes: Humanizing the Birth Process

The motives that led the participants to draw up a BP coincided with those posed in the ‘Estrategia de Atención al Parto Normal’, as well as with the recommendations from the WHO and the Spanish Society of Gynecology and Obstetrics (SEGO) regarding attention given in childbirth, which mention improving humane aspects and promoting the respect for the physiological process with minimum intervention [9,17]. Whitford et al. reflected that some mothers saw it as a positive step, in order to guarantee their preferences and wishes [18]. The participation of women in the decision-making process during birth is essential for managing stress and providing maternal support, as well as for the psychosocial preparation of the expectant mother [19]. As was stated by the women in this study, other authors also concluded that the BP document may provide a means to improve communication between mothers and midwives [20]. Other authors considered that it represented an opportunity to improve the women’s trust in the medical staff [21]. Hildingsson concluded that factors which influenced the birth experience included expectations of the birth, information from the medical staff, communication with the staff and control of the process, among others [22].

### 4.2. Category 2: Information and Primary Care

Although the prenatal classes included all the aspects related to pregnancy, birth and the postpartum period, the fact that the medical staff involved appeared to be up-to-date with the latest developments and were willing to adapt to the needs of modern women were valued positively [23].

As with our participants, Gibeau stated that women showed greater dissatisfaction if the medical professional giving the classes was not familiar with the practice of making BPs [24]. Anderson et al. compared standardized and personal BPs, and considered that standardized BPs fostered better communication with the medical staff, and could be an important communication tool by facilitating a discussion between women and their health providers about their wishes and expectations [23]. Some authors acknowledged that health information on BPs is not available to all women, and that having a high level of obstetric risk and a high number of births are independent factors in not receiving such health information [25].

### 4.3. Category 3: Expectations Regarding the Care Received

Most of the studies link the satisfaction of the birth experience with the women’s previous expectations, and the extent to which these were fulfilled [11]. Many authors also related the use of the BP to higher levels of satisfaction [20,23,26]. Anderson et al. found that most women reported using a BP with a subsequent birth, suggesting that most patients found that the BP had a positive effect [23]. In contrast, other authors such as Jolles et al. concluded that the women who presented a BP did not report high levels of satisfaction in the postpartum period [14], or felt less satisfied [27]. In fact, Higuero-Macías et al. even concluded that women who presented a BP displayed a greater degree of dissatisfaction, regardless of the extent to which it was fulfilled [28]. In this study, however, the degree of dissatisfaction was closely related to a complete lack of fulfilment of the expectations, whilst the partial or total fulfilment was linked to a very satisfactory experience. Medeiros et al. also found that unrealistic expectations of the women with their BP may cause dissatisfaction with the birth experience [13].

#### Subcategory 3.1: Demanding for Improvements Immediately after Birth

The women acknowledged the benefits of the immediate initiation of breastfeeding, with the guidance and support of the medical staff, as well as not bathing, weighing and measuring the baby, etc., immediately, and so giving greater importance to immediate skin-to-skin contact [9]. In a study on the influence of BPs on the expectations and satisfaction of mothers, both breastfeeding and the establishing of skin-to-skin contact were issues that highly interested the mothers [28]. In another study, the authors found a higher rate of allowing skin-to-skin contact among the mothers that presented a BP [3]. As in our study, Paz Pascual et al. found that the women’s opinions regarding the care received during the postpartum period was rather negative [29].

### 4.4. Category 4: Results of Using the Birth Plan

Most participants found the BP useful as a way of acquiring information about the birth process and as an initial contact with the experience of labor. However, other authors concluded that the BP did not improve the experience of birth, but it had beneficial effects with regard to fear and pain [30]. In a review by Medeiros et al., the authors concluded that the benefits of using the BP included the promotion of natural and physiological birth processes, better communication with the medical staff, increased knowledge about the birth process, a greater sense of control and autonomy, better obstetric and neonatal outcomes and a higher degree of maternal satisfaction [13]. Some authors concluded that a BP could influence the mode of birth, and be associated with a higher rate of vaginal delivery [3,5,12].

#### Subcategory 4.1: A Tool for Providing Legal Advice and Peace of Mind

Fernández-Arroyo draws attention to the term “female empowerment”, with which he refers to the increased knowledge and self-confidence acquired by women who completed BPs in different aspects relating to pregnancy and labor [31]. This document can also act as a legal tool, as it represents a genuine ‘informed consent’ form which expresses the patient’s wishes and is supported by the relevant legislation [32]. It should be noted that BP is a document of prior instructions, and therefore may have legal consequences due to the obligation of the professionals to respect what is expressed in it; however, the birth is a dynamic and unpredictable process, whose results can create frustration for the woman [33].

Mouta et al. also considered the BP as a tool for increasing the empowerment of women in the birth process [34]. According to the results of the present study, having more information gave the women greater peace of mind. However, BPs are not used by many women. López-Gimeno et al. concluded that the main reason given by women for not presenting a BP was because the hospital midwives did not read them [7]. In this sense, López-Toribio et al. considered that BPs were experienced as an insufficient tool to promote women’s participation in decision-making, as the professionals themselves rejected their use [35].

### 4.5. Impact the COVID-19 Pandemic on Birth Plans

Although few studies have examined this issue, the lack of material and personnel resources, crowded hospitals, overworked staff and other limitations due to the COVID-19 pandemic have changed health systems in general and, in particular, the obstetric services with implications for pregnant women and their BPs. Thus, some authors concluded that 45% of women altered some aspect of their birth plans because of COVID-19, and reported a much higher preference for out-of-hospital births [36]. Further studies are needed to identify how the pandemic is affecting BPs.

The results obtained in our study should be taken with caution, due to the limitations of the study. The most important limitation was the sample size, which was low because some women chose not to take part in the study, although the numbers were sufficient to cover all of the study categories. In addition, the study was limited to women who presented a BP at a tertiary hospital, and adherence to the BP was very low.

## 5. Conclusions

Having a BP provides women with greater knowledge of the process of labor and birth, and it helps them to express their wishes, which are generally related to humanizing the process and experiencing it in the most natural way possible. It is considered as a good communication tool with the medical staff, although greater involvement of the staff is needed, and they should be better trained and more aware of the issues involved. In primary care, there is little information available regarding BPs, and so mothers find information from sources outside the health environment. The degree of satisfaction of these women is related to the level of fulfillment of their expectations. This compliance is influenced by the circumstances of the labor, and the degree of involvement of the healthcare professionals. The women also requested greater care after the birth: in this survey, skin-to-skin contact and the rapid initiation of breastfeeding were two important sources of dissatisfaction. In general, they experienced an empowerment of their own role within the birth process, were able to enforce their own decisions, and were aware of the legal value of the Birth Plan.

Implications for the clinical practice: the BP should be considered a useful document in childbirth care. These authors recommend the active participation of women in the process of childbirth, and believe that BPs improve women’s levels of satisfaction with the experience of giving birth, and the communication between the mother and the health staff. In this regard, it is the responsibility of midwives in all primary care centers to provide mothers-to-be with accurate, up-to-date information about the childbirth process.

## Figures and Tables

**Table 1:** Analysis Categories.

Categories		Subcategories
Category 1	Respecting the woman’s wishes: Humanizing the birth process.	BP: a means of encouraging better communication.
Category 2	Information and Primary Care.	
Category 3	Expectations regarding the care received.	Demanding for improvements immediately after birth
Category 4	Results of using the BP.	A tool for providing legal advice and peace of mind.

## Data Availability

The data presented in this study are available on request from the corresponding author.
